# Efficiency of Recovery of the Bioactive Principles of Plants by Comparison between Solid–Liquid Extraction in Mixture and Single-Vegetable Matrices via Maceration and RSLDE

**DOI:** 10.3390/plants12162900

**Published:** 2023-08-09

**Authors:** Daniele Naviglio, Marco Trifuoggi, Francesca Varchetta, Viviana Nebbioso, Angela Perrone, Laura Avolio, Eleonora De Martino, Domenico Montesano, Monica Gallo

**Affiliations:** 1 Department of Chemical Sciences, University of Naples Federico II, Via Cintia, 4, 80126 Naples, Italy; naviglio@unina.it (D.N.); marco.trifuoggi@unina.it (M.T.); fravar87@gmail.com (F.V.); viviana.nebbioso@gmail.com (V.N.); an2016a@libero.it (A.P.); lauraavolio02@gmail.com (L.A.); eleonorademartino97@gmail.com (E.D.M.); 2 Department of Research & Development, Erbagil s.r.l., Via L. Settembrini 13, 82034 Telese Terme, Italy; domenico_montesano@yahoo.it; 3 Department of Molecular Medicine and Medical Biotechnology, University of Naples Federico II, Via Pansini 5, 80131 Naples, Italy

**Keywords:** medicinal plants, herbal preparations, natural products, phytocompounds, beneficial effects, antioxidant activity, RSLDE, solid–liquid extraction, Naviglio extractor

## Abstract

The term “officinal” derives from the Latin and includes all medicinal, aromatic and perfume plant species, which have long been a subject of interest for multiple purposes: health, food, pharmacological, cosmetic and so on. In this work, a study on six different species of medicinal plants, particularly characterized by digestive, choleretic and diuretic properties, was carried out: rosemary (*Rosmarinus officinalis*), sage (*Salvia officinalis*), laurel (*Laurus nobilis*), gentian (*Gentiana lutea*), dandelion (*Taraxacum officinale*) and rhubarb (*Rheum palmatum*). The roots and aerial parts of plants were separately extracted with two different techniques—maceration and rapid solid–liquid dynamic extraction (RSLDE)—and the quali/quantitative analysis of active ingredients have been determined by applying dry residue, Folin–Ciocalteu and DPPH assays. Data obtained have provided useful answers regarding the efficiency of the extraction carried out on a mixture or on single plants, allowing us to evaluate the best choice according to the cases and the final uses.

## 1. Introduction

The use of medicinal plants is very ancient and, in fact, for many centuries, they were used as the only available medical remedies [1,2]. Fortunately, many plants can supply active ingredients widely used by the pharmaceutical industry (cardiotonics, salicylic acid, anticancer drugs, etc.), contributing to human wellbeing and health. In fact, medicinal plants are characterized by the presence of certain classes of active ingredients, such as polyphenols, glycosides and tannins, that could be a natural aid to illnesses. For example, based on the chemical structure, polyphenols are divided into ten or more different groups, but the main classes are stilbenes, lignans, flavonoids and phenolic acids [3]. Moreover, the flavonoids can then be divided into further classes, among which it is possible to find isoflavones and anthocyanins. These substances have various properties: first of all, their chemical structure allows them to function as antioxidant molecules and therefore to protect the plant from oxidative stress and free radicals. However, their intake also determines numerous beneficial effects on human health, as demonstrated by numerous studies [4,5,6,7]. The chemical compounds contained in plants, and which positively act on the human (or animal) organism, are called active principles, responsible for some specific biological activity. They are very varied, including polyphenols, alkaloids, flavonoids, glycosides, saponins, tannins and essences, and are often localized or abundant in certain plant tissues. Chemical compounds naturally present in plants are defined as phytochemicals; some are responsible for the organoleptic properties, color and smell of the plant, while others have a biological function as a defense mechanism against parasites or other organisms. The phytocomplex constitutes the set of all the molecules present in the plant drug, the so-called “active ingredients”, together with other non-active components, but which are equally important for the plant. The active ingredients are stored in the different parts of the plant (leaves, flowers, roots, seeds) and are responsible for the medicinal properties of the plant itself. In plants, the synthesis and accumulation of these active ingredients is a very complex process, influenced by numerous factors, both of a genetic nature and by external environmental factors (light, temperature, water, salinity, etc.) [8]. These compounds, generally, are called secondary metabolites, and have they specific functions for plant survival. Secondary metabolites can mainly be divided into three families of natural compounds: alkaloids, phenolic compounds (polyketides and phe-nylpropanoids) and terpenoids. The period in which the plants are harvested is also fundamental: the most suitable period (balsamic period) is the one in which the parts are richest in active substances, and it almost always coincides with flowering. Almost all drugs can be preserved and the most common method to keep them in good condition is that of hot drying, which is all the better the faster the more complete it is; moreover, this method is cheaper than the others. Alternatively, freeze drying is also often employed but with a higher cost. Furthermore, a single plant may contain several active substances (biologically active secondary metabolites); consequently, it can have different healthy properties [9,10]. Therefore, the extraction methods of the active principles from plants and vegetables are of great importance and differ depending on whether the fresh or dried plant is used [11,12,13]. The phytotherapeutic preparations from fresh plants are, for example, essential oils, juices, glyceric macerates and mother tinctures, while those from dried plants are extracts, herbal teas and powders. The choice of the extraction method, the operating conditions and the solvents is of fundamental importance to obtaining the active ingredients in a qualitative and quantitative measure [14,15]. This is often not an easy operation and is the result of a careful compromise between the numerous characteristics of the compounds themselves in order to avoid extracting unnecessary plant material as well, such as chlorophylls, lipids, waxes, etc. Depending on the type of extraction procedure chosen, the concentrations of the individual active ingredients can vary, and consequently, the same plant can be used for different purposes and applications. On the other hand, different plants can have similar but also different actions, and their simultaneous use can enhance their effectiveness (synergy of the phytocomplex). In other words, a mixture of plant substances shows greater biological activity and offers optimal synergistic effects (entourage effect) compared to the action of the individual constituents. The solid–liquid extraction techniques are divided between conventional and innovative. The latter, of more recent construction, generally have greater efficiency and a lower environmental impact [16,17]. In particular, the extracts were obtained by solid–liquid extraction, a process that allows us to separate one or more components present in a solid phase (food, vegetable or animal type matrices) using a liquid phase (extracting or carrier liquid) [18,19,20]. The most used solvents in herbalist tradition are water, ethyl alcohol, glycerin, and their mixture, wine and oil; other organic solvents are acetone and ether. The first phase of the extracting process consists of crushing the solid matrix to favor the diffusion of the solvent inside it, increasing the superficial area in contact with liquid; in the second phase, wetting, the solvent diffuses, occupying all the accessible spaces of the matrix; in the third phase, the various active ingredients pass into the solvent due to diffusion (Fick’s law) and osmosis, creating a solution of a certain concentration. To facilitate the extraction process, a much larger volume of solvent is used than that of the solid phase, and it is often assisted by raising the temperature. These extraction techniques essentially exploit diffusion and osmosis, two relatively slow phenomena, to migrate the principles dissolved in the solvent towards the outside of the solid until equilibrium is reached. Once this equilibrium is reached, the solution rich in extracted compounds is separated by physical processes of filtration and centrifugation. Many different parameters can influence the extraction yield, such as the size and physical state of the matrix particles, the temperature, the solvent/matrix ratio and the relative contact times [21,22]. In this work, a comparison between two solid–liquid extraction techniques—conventional maceration and the innovative RSLDE—was carried out in order to evaluate the differences in the extraction efficiency of the bioactive compounds obtained from the plants extracted in mixture or individually and, consequently, to establish the extraction methods according to the different fields of application of the extracts obtained.

## 2. Results and Discussion

### 2.1. Maceration vs. RSLDE

The natural products of medicinal plants, both as pure compounds and as standardized extracts, due to their chemical diversity, offer numerous opportunities for use in various fields of human and animal applications. Thus, interest in edible plants in particular has grown worldwide due to the presence of plant extracts of various types of bioactive compounds and their proven beneficial effects on health [23,24,25]. However, since extraction is the most important step in the analysis of the constituents present in plant matrices, the strengths and weaknesses of two extraction techniques, such as maceration and RSLDE, have been discussed in this work, but above all, for the first time, the effects of the extraction carried out on a mixture of plants were evaluated compared with a mixture made up of single extracts from the same plants.

Maceration is one of the oldest and simplest techniques. The extraction process is generally characterized by a long period of extraction: two or three weeks to exhaust the plant. The Official Pharmacopoeia—that is, the reference text for the preparations in herbalists’ sector—specifies twenty-one days to obtain the major parts of extracts from medicinal plants, with occasional mixing of the maceration batch. The diffusion and osmosis processes used in this extraction are speeded up through the use of ultrasound or microwaves or through an increase in temperature so as to act on the kinetic energy of the molecules of the solid [26]. Furthermore, to ensure the diffusion of the extracted substances throughout the mass of the extracting liquid, it is necessary to agitate the system and remove the micro-equilibrium established near the solid matrix, in this way avoiding the premature stopping of extraction phenomenon. Among the disadvantages of this technique, in addition to the long times required and it not always being compatible with the properties of the matrix, there is the incomplete extraction of the matrix itself and the non-reproducibility of the extract content (standardized extract) because this type of solid–liquid extraction can be defined as passive extraction. Furthermore, the vegetable matrices cannot be macerated in water as they undergo degradation processes. However, it is still a valid and in-use technique; in fact, in a recent work, an innovative method of successive macerations was proposed using a mixture of solvents with the aim of simultaneously improving the yield, the distribution of the compounds between the different phases and reducing the volume of extraction solvents [27]. On the other hand, the most recent extraction techniques try to balance a series of factors, such as the quality of the product obtained, the efficiency of the process, the production costs and a low environmental impact [28,29,30]. From this point of view, RSLDE combines all these factors, in fact, it is an innovative technique which allows for extraction at room temperature in a short time with a reduced environmental impact (green) [31]; this kind of solid–liquid extraction can be defined as an active process because compounds are forced to exit from the inner of vegetable thanks to difference of pressure. The Naviglio extractor works by alternating a static phase, in which the pistons present in it push simultaneously on the liquid causing an increase in pressure, with a dynamic phase, during which the pistons are moved from their equilibrium position, and there is an alternation of thrusts between the two pistons themselves, with a reduction in pressure and the generation of a mixture of the liquid throughout the system to diffuse substances and thus to reduce the concentration around the solid matrix. It is at this moment that the extraction of the solid matrix takes place, made possible by a difference in pressure between the inside and the outside of the sample. The extractable substances, not chemically bonded to the principal structure of the solid matrix, at each extraction cycle, are dragged out by a mechanical effect. The dynamic phase also allows for the rapid and complete mixing of the solid matrix and the instantaneous diffusion of the extracted substances throughout the mass of the liquid, avoiding supersaturation phenomena around the solid that could stop the extractive process. This system makes this technique effective both in terms of extraction time and recovery efficiency and the quality of extract of the active ingredients contained in the plant matrix, as demonstrated by its use in various fields of application, e.g., herbal, pharmaceutical, cosmetic and food [32,33,34,35,36,37].

### 2.2. Leaf Extraction

**Figure 1:**
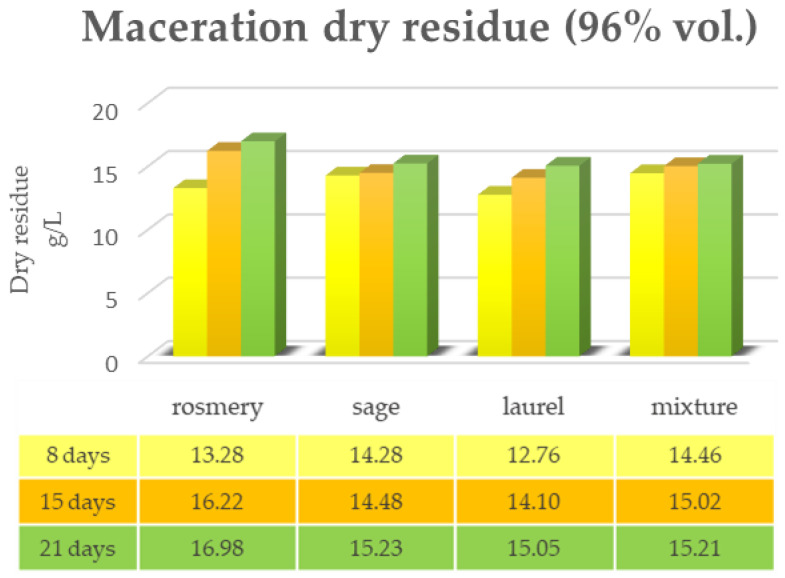
Determination of the dry residue after extraction via maceration with 96% ethyl alcohol carried out on rosemary, sage and laurel leaves individually and in mixtures.

**Figure 2:**
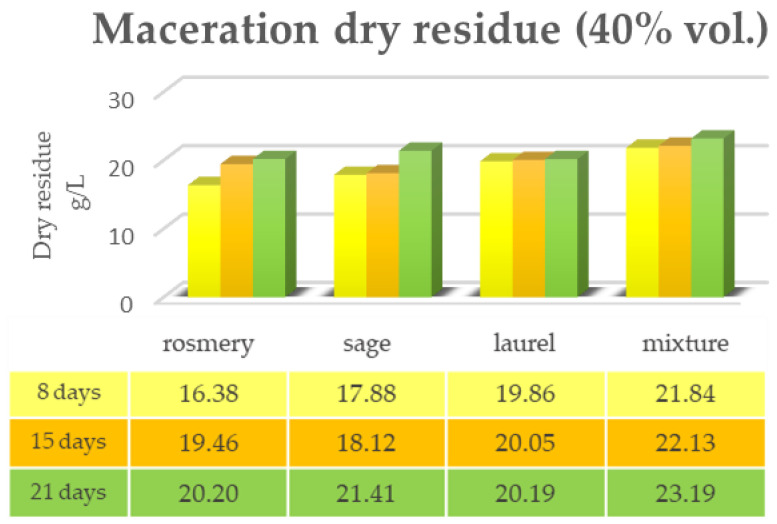
Determination of the dry residue after extraction via maceration with a 40% hydroalcoholic solution carried out on rosemary, sage and laurel leaves individually and in mixtures.

**Figure 3:**
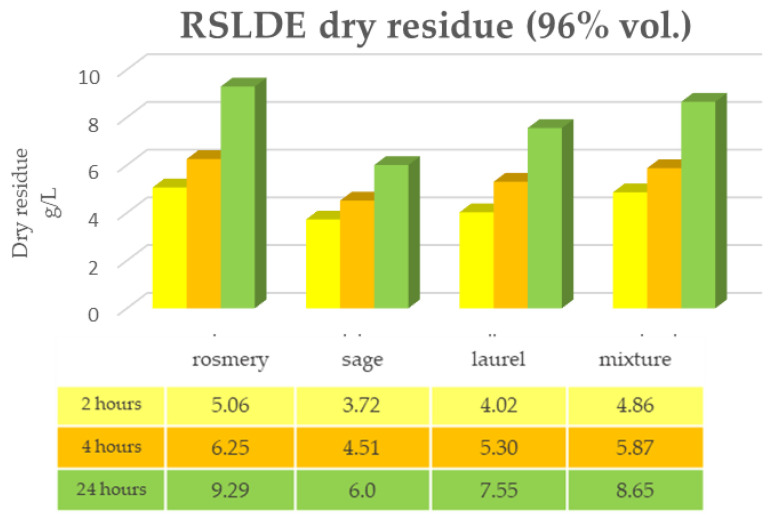
Determination of dry residue after extraction via RSLDE with 96% ethyl alcohol carried out on rosemary, sage and laurel leaves individually and in mixtures.

**Figure 4:**
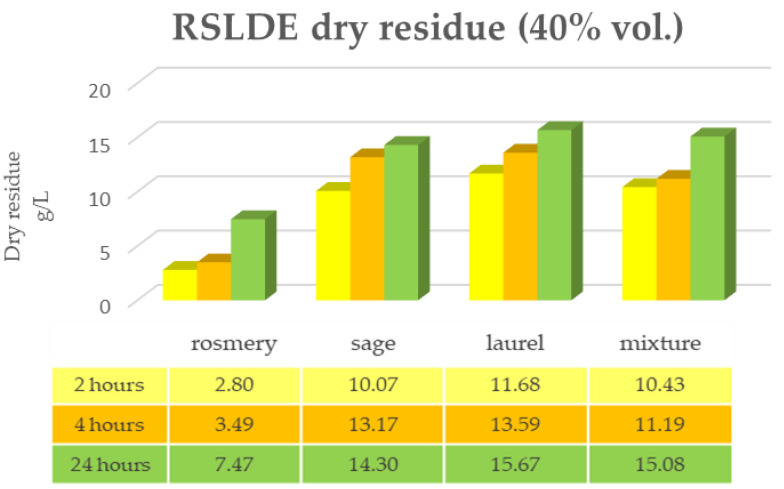
Determination of dry residue after extraction via RSLDE with 40% hydroalcoholic solution carried out on rosemary, sage and laurel leaves individually and in mixtures.

Figure 1 and Figure 2 show the data of the dry residue obtained from the extraction via the maceration of the leaves of the first triad of plants (rosemary, sage and laurel) extracted individually and in mixtures, both in alcoholic solution (ethyl alcohol 96% vol.) and in hydroalcoholic solution (40% vol.). The same determination was performed under the same conditions via RSLDE (Figure 3 and Figure 4).

As can be seen from Figure 1, Figure 2, Figure 3 and Figure 4 the comparison of the data shows that the dry residue, expressed in g/L, increases with the passing of the days as regards the extraction during maceration, while for the extracts using RSLDE, the increase is a matter of hours.

**Figure 5:**
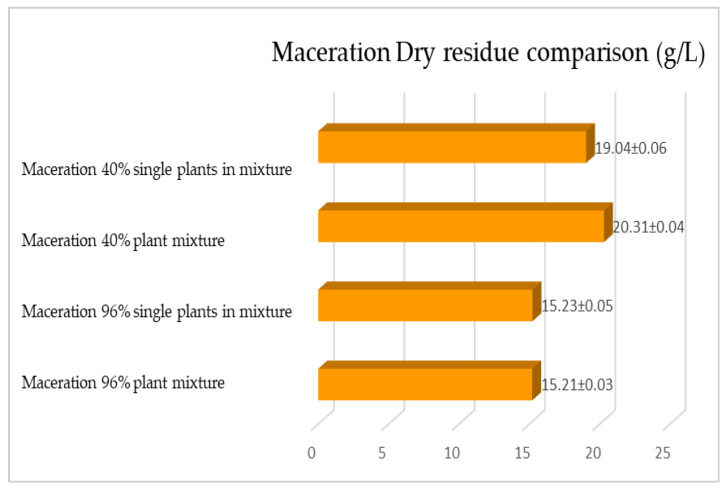
Comparison between the dry residue values obtained by macerating rosemary, sage and laurel leaves individually and mixed in the two different solvents. Each bar represents the mean ± SD of three independent experiments.

**Figure 6:**
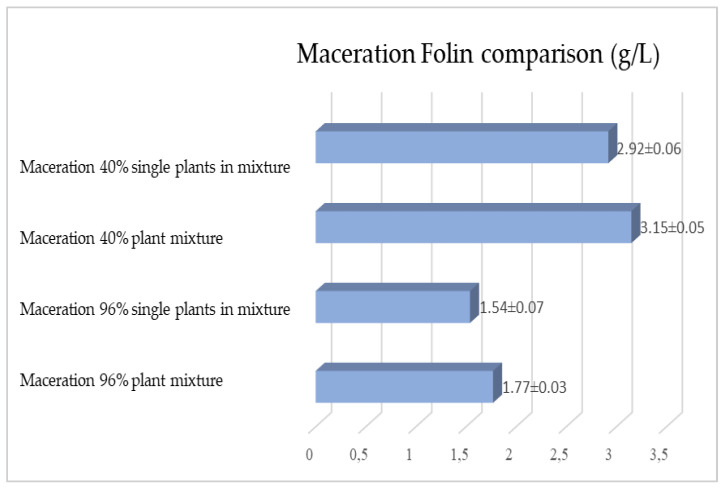
Comparison between the polyphenol concentration values obtained via the maceration of rosemary, sage and laurel leaves individually and in mixtures in the two different solvents. Each bar represents the mean ± SD of three independent experiments.

**Figure 7:**
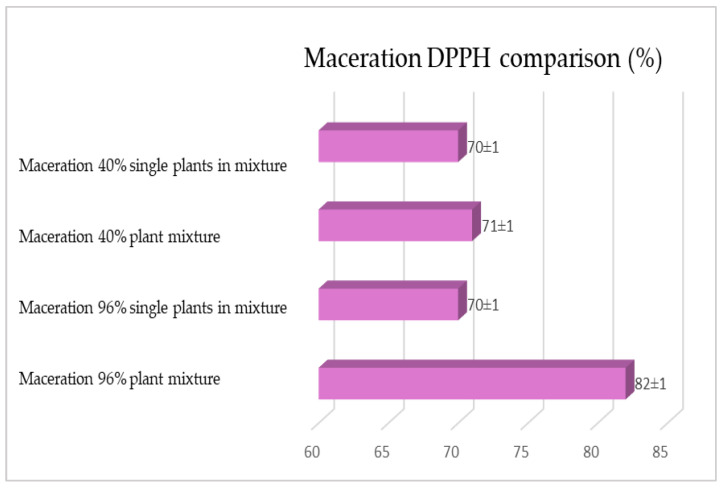
Comparison between the antioxidant activity values obtained via the maceration of rosemary, sage and laurel leaves individually and mixed in the two different solvents. Each bar represents the mean ± SD of three independent experiments.

Figure 5, Figure 6 and Figure 7 show the comparison of the data obtained from the determination of the dry residue, the concentration of polyphenols and the antioxidant activity via extraction via maceration from the mixture composed of a triad of rosemary, sage and laurel and from the mixture of the individual macerates of the same plants in the same proportions in the two different solvents.

**Figure 8:**
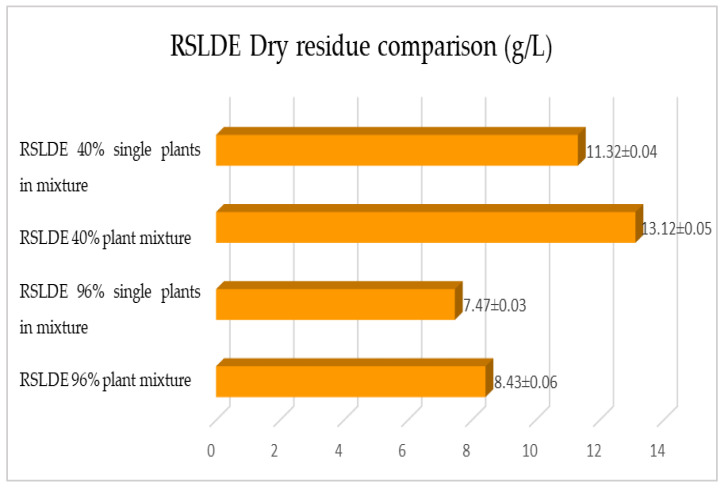
Comparison of dry residue values obtained via RSLDE from rosemary, sage and laurel leaves individually and in mixtures in the two different solvents. Each bar represents the mean ± SD of three independent experiments.

**Figure 9:**
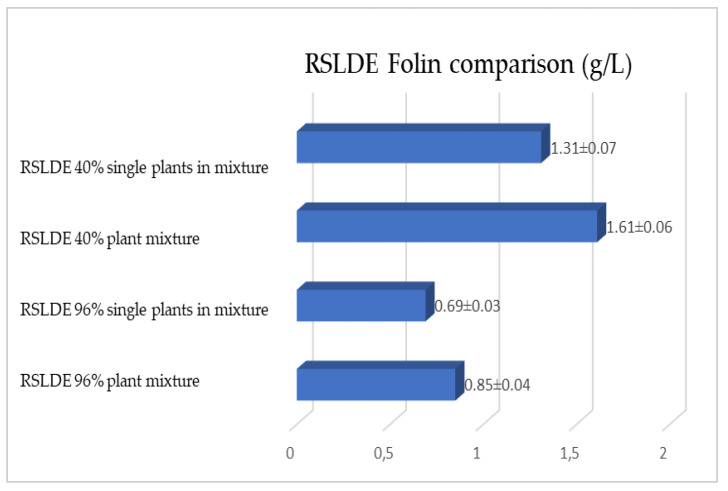
Comparison of polyphenol concentration values obtained via RSLDE from rosemary, sage and laurel leaves individually and in mixtures in the two different solvents. Each bar represents the mean ± SD of three independent experiments.

**Figure 10:**
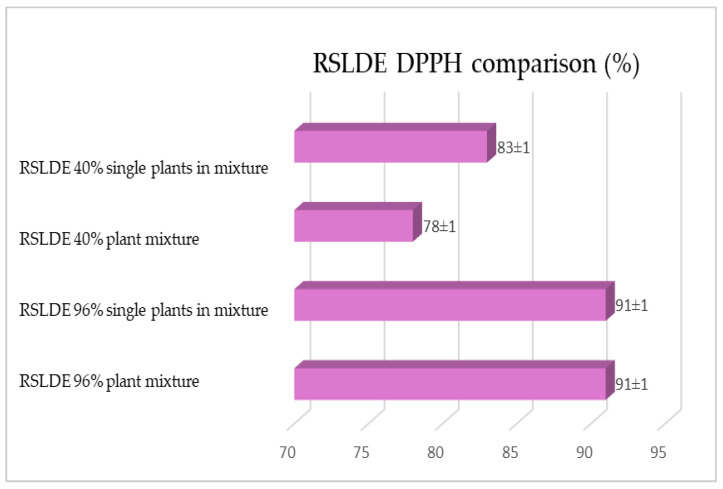
Comparison of the antioxidant activity values obtained via RSLDE from rosemary, sage and laurel leaves individually and in mixtures in the two different solvents. Each bar represents the mean ± SD of three independent experiments.

The same comparison was made for plants extracted via RSLDE. Figure 8, Figure 9 and Figure 10 show the comparison of the data obtained via the determination of the dry residue, the concentration of polyphenols and the antioxidant activity by means of RSLDE from the mixture composed of a triad of rosemary, sage and laurel and from the mixture of the single macerates of the same plants in the same proportions in the two different solvents.

It is possible to highlight from Figure 5, Figure 6, Figure 7, Figure 8, Figure 9 and Figure 10 that the mixture of this first triad of plants in both solvents shows a higher concentration in terms of dry residue, polyphenol concentration and antioxidant activity, i.e., 10–15% higher than the mixture prepared by mixing the single extracts via maceration and the single extracts via RSLDE in the same proportions.

### 2.3. Root Extraction

**Figure 11:**
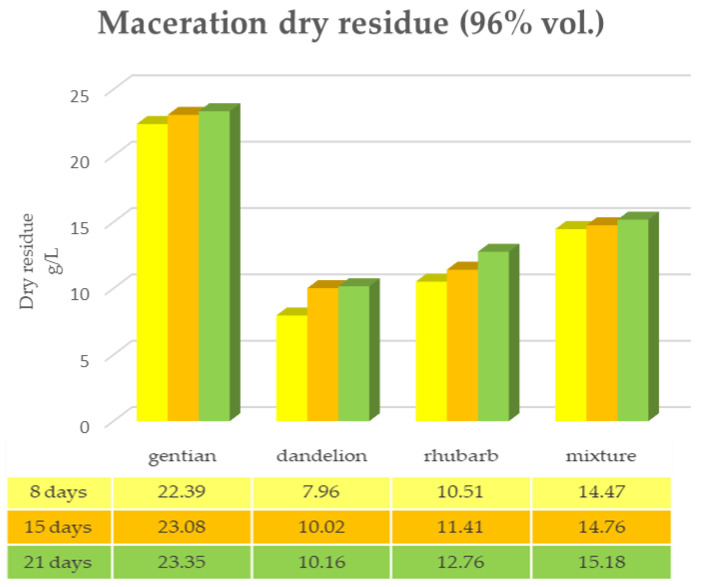
Determination of the dry residue after extraction via maceration with 96% ethanol carried out on the roots of gentian, dandelion and rhubarb individually and in mixtures.

**Figure 12:**
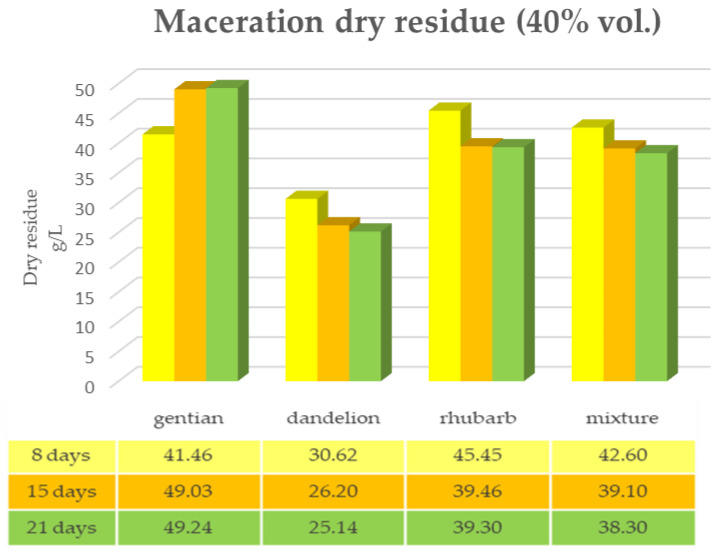
Determination of the dry residue after extraction via maceration with a 40% hydroalcoholic solution carried out on the roots of gentian, dandelion and rhubarb individually and in mixtures.

**Figure 13:**
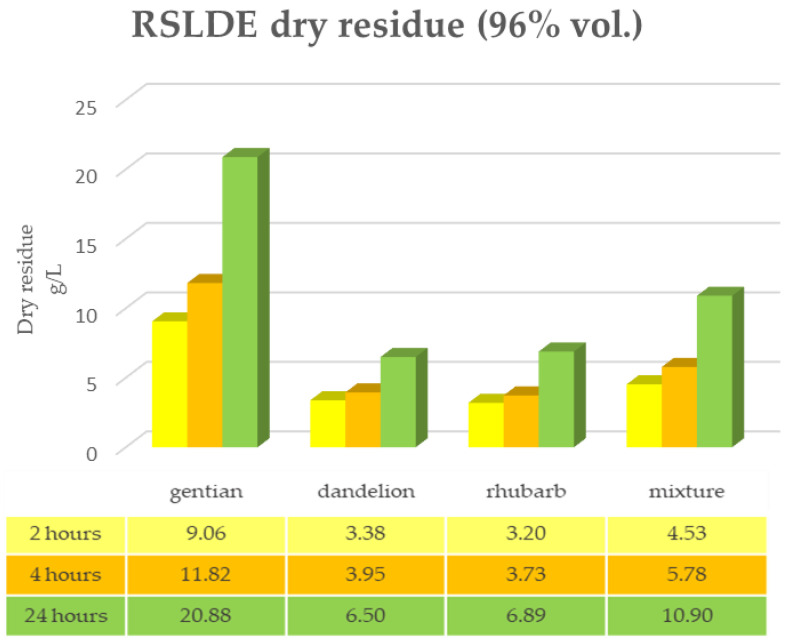
Determination of the dry residue after extraction via RSLDE with 96% ethanol carried out on the roots of gentian, dandelion and rhubarb individually and in mixtures.

The same determinations reported previously were carried out on the second set of gentian, dandelion and rhubarb plants, whose matrix extracted in these cases is represented by the roots. Figure 11, Figure 12 and Figure 13 show the comparison of the data obtained via the determination of the dry residue, the concentration of polyphenols and the antioxidant activity via extraction via maceration from the mixture composed of the triad of gentian, dandelion and rhubarb and from the mixture composed of individual macerates of the same plants in the same proportions in the two different solvents: alcoholic solution (96% vol.) and hydroalcoholic solution (40% vol.).

**Figure 14:**
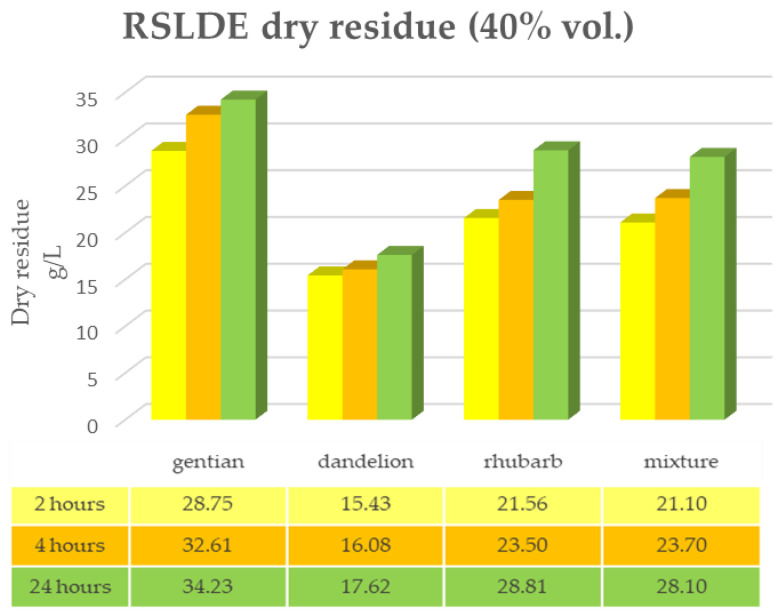
Determination of the dry residue after extraction via RSLDE with 40% hydroalcoholic solution carried out on the roots of gentian, dandelion and rhubarb individually and in mixtures.

As can be seen from Figure 11, Figure 12, Figure 13 and Figure 14, in this case also, the comparison of the data shows that the dry residue, expressed in g/L, increases with the passing of the days as regards the extraction during maceration, while for the extracts using RSLDE, the increase a matter of hours.

Furthermore, in the extraction via maceration in a hydroalcoholic solution (40% vol.), there is a decrease in the value of the dry residue as regards dandelion and rhubarb, probably due to a degradation of the matrix after a maceration period of more than 10–15 days.

**Figure 15:**
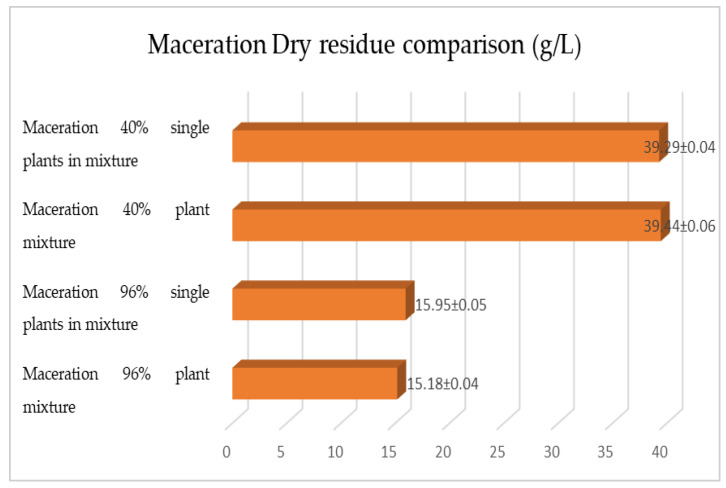
Comparison between the dry residue values obtained by macerating the roots of gentian, dandelion and rhubarb individually and mixed in the two different solvents. Each bar represents the mean ± SD of three independent experiments.

**Figure 16:**
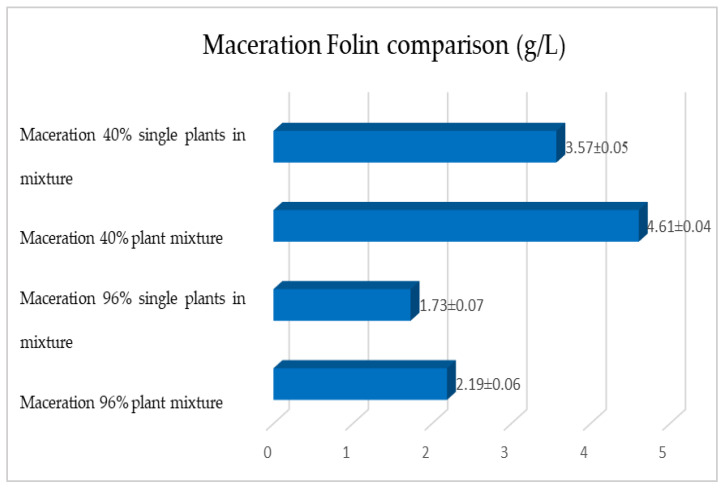
Comparison between the polyphenol concentration values obtained via maceration from the roots of gentian, dandelion and rhubarb individually and as a mixture in the two different solvents. Each bar represents the mean ± SD of three independent experiments.

**Figure 17:**
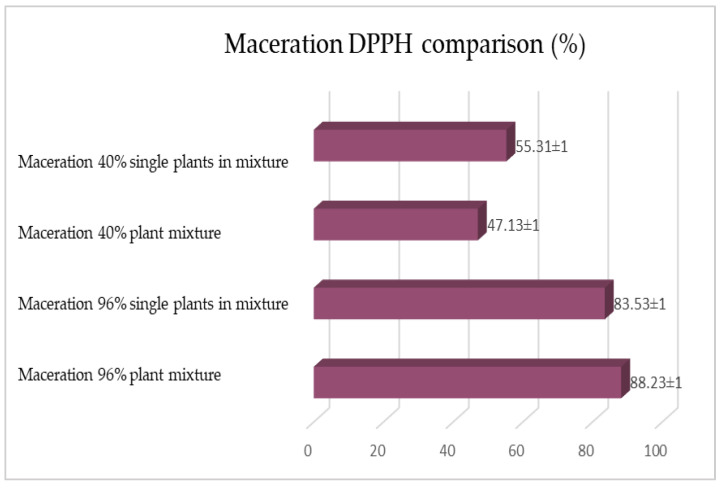
Comparison between the antioxidant activity values obtained via maceration from gentian, dandelion and rhubarb roots individually and mixed in the two different solvents. Each bar represents the mean ± SD of three independent experiments.

Figure 15, Figure 16 and Figure 17 show the comparison of the data obtained via the determination of the dry residue, the concentration of polyphenols and the antioxidant activity via extraction via maceration from the mixture composed of the triad of gentian, dandelion and rhubarb and from the mixture of the individual macerates of the same plants in the same proportions in the two different solvents.

**Figure 18:**
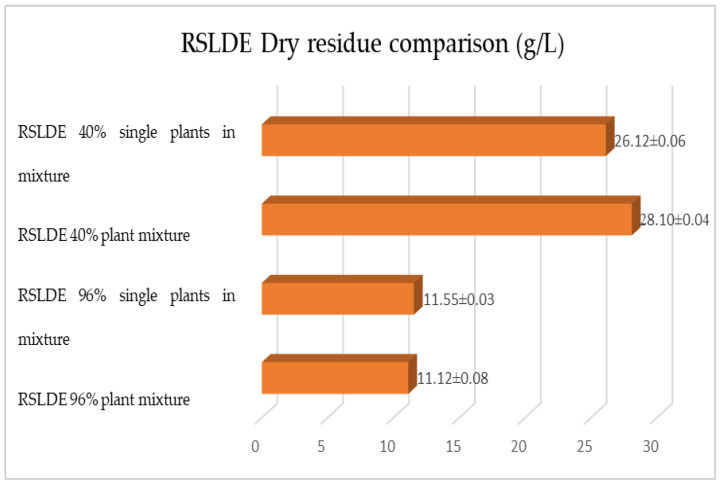
Comparison of dry residue values obtained via RSLDE from gentian, dandelion and rhubarb roots individually and in mixtures in the two different solvents. Each bar represents the mean ± SD of three independent experiments.

**Figure 19:**
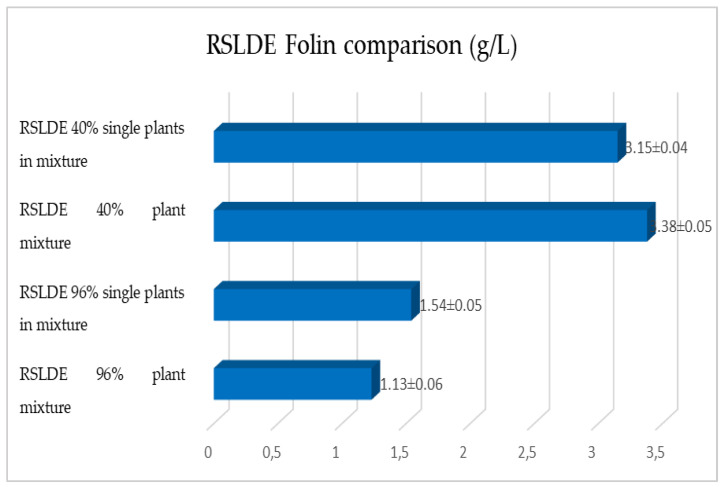
Comparison of polyphenol concentration values obtained via RSLDE from gentian, dandelion and rhubarb roots individually and in mixtures in the two different solvents. Each bar represents the mean ± SD of three independent experiments.

**Figure 20:**
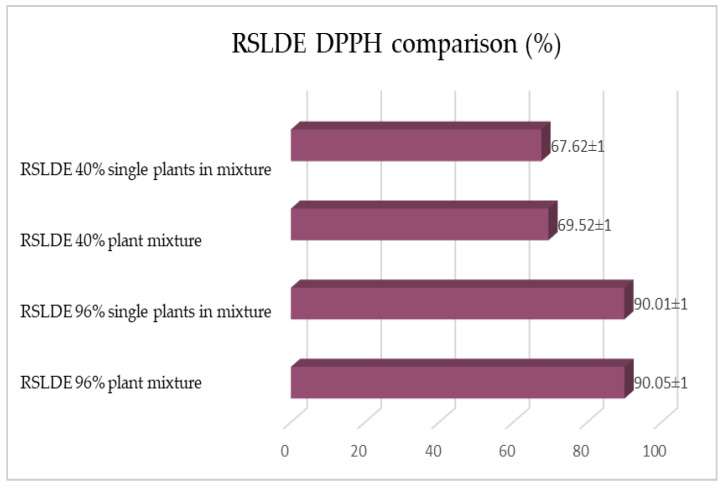
Comparison of the antioxidant activity values obtained via RSLDE from gentian, dandelion and rhubarb roots individually and in mixtures in the two different solvents. Each bar represents the mean ± SD of three independent experiments.

As previously reported, the same comparison was made for plants extracted via RSLDE. Figure 18, Figure 19 and Figure 20 show the comparison of the data obtained via the determination of the dry residue, the concentration of polyphenols and the antioxidant activity by means of RSLDE from the mixture composed of the triad of gentian, dandelion and rhubarb and from the mixture of the individual macerates of the same plants in the same proportions in the two different solvents.

The comparison of the results obtained shows that also for this second triad of plants, the mixed extraction of the three plants turns out to be better performing, with a variation of the order of 10–15% more than the mixture prepared by mixing the individual extracts in the same proportions of the mixture and carrying out the appropriate determinations. In order to obtain a more correct evaluation of the antioxidant activity of the obtained extracts, a comparison was made with another method, i.e., the FRAP assay, which is based on the ferrous-reducing activity of the antioxidant compounds. The results obtained showed that the values for the FRAP ranged from 13.65 to 79.89 mg TE/g. Moreover, in this case (in both series of the three plants), the results of the antioxidant activity of the three plants in the mixture are higher than in the preparation obtained by mixing the single extracts in the same proportions of the mixture.

In summary, although further studies are underway to identify and quantify the bioactive compounds present in the various extracts obtained, two extraction methods were compared in this study to obtain bioactive compounds from two different plant parts, i.e., the leaves and roots. The experimentation conducted in this work is part of a larger project which includes instrumental analytical determinations for deep chemical characterization and biological assays on cell lines to evaluate their antioxidant and anti-inflammatory properties. In this first phase, attention was focused on the effectiveness of the extraction method and on the innovation of the extraction. The results obtained will enable the use of the innovative extraction method with proven efficacy in order to guarantee the best quality of the extract. On the other hand, the choice of the matrix to be extracted depends on both the type of plant and on the part of the plant with the higher content of bioactive compounds or the greater interest [38]. Furthermore, to the best of our knowledge, this is the first study in which the extraction efficiency of plants taken individually was compared with those mixed of other plants to evaluate the differences. Therefore, a conventional extraction method, i.e., maceration, was used and compared with an innovative one, i.e., RSLDE, which allows a faster, more efficient, but above all, greener extraction, while also allowing for the recovery of the solvent used. On the other hand, RSLDE can be considered, to all intents and purposes, a “green technique” as it operates at room temperature and with minimal use of solvent and minimal energy. Among other things, the extraction solvent can be recovered and reused in line with the current principle of the circular economy. In fact, several applications of RSLDE in various sectors are reported in the literature with respect to both conventional techniques and innovative ones, in which its efficiency is highlighted [39].

## 3. Materials and Methods

### 3.1. Plant Matrices

The medicinal plants used were as follows: rosemary, sage, laurel, gentian, dandelion and rhubarb. They were supplied by the ARDA NATURA company (Arda Natura Srl, Fiorenzuola D’Arda, Piacenza, Italy). All reagents and solvents were analytical grade and purchased from Carlo Erba (Milan, Italy), Sigma-Aldrich (Saint Louis, MO, USA) and Fluka (Buchs, Switzerland), as ethyl alcohol 96% (*v*/*v*), Folin–Ciocalteu reagent and 2,2-diphenyl-1-picrylhydrazyl (DPPH) gallic acid as standard.

In the mixed extractions (1:1:1), the matrix used in the first triad were as follows: rosemary, sage and laurel plants were represented by the dried and chopped leaves; while in the case of the second triad—made up of gentian, dandelion and rhubarb—by the dried and shredded roots. The solvents used to extract the active ingredients were ethyl alcohol (96% *v*/*v*) and a hydroalcoholic solution (40% *v*/*v*).

The macerates of the 6 single plants in the 2 different solvents were prepared by weighing 50 g of each single plant, while 51 g (17 g of rosemary, 17 g of sage and 17 g of laurel) were used for the mixtures (part used: leaves), and the same were used for the mixture prepared with gentian, dandelion and rhubarb (part used: roots).

Maceration: The plants were placed in a closed glass container with 500 mL of solvent and kept in the dark for 21 days (Official Farmacopoeia), occasionally shaking. Therefore, at intervals of 2, 4 and 24 h, 15 mL of extract were taken, which was then filtered on filter paper and used to determine the dry residue, the yield and the concentration of polyphenols in g/L by assay with Folin–Ciocalteu reagent and antioxidant activity by DPPH assay. Lastly, 15 mL of fresh solvent were added.

RSLDE: The extractions of the individual plants and their respective mixtures were extracted through the use of the Naviglio extractor, which allows for faster and more efficient extraction. The vegetable matrices and solvents were used in the same quantities and volumes of the extraction by maceration (50 or 51 g in 500 mL). Therefore, at intervals of 2, 4 and 24 h, 15 mL of extract were taken, which was then filtered on filter paper and used to calculate the dry residue, the yield and the concentration of polyphenols in g/L via assay with Folin–Ciocalteu reagent and antioxidant activity via DPPH assay. Lastly, 15 mL of fresh solvent were added.

The dry residue was obtained by drying exactly 10 mL of the sample in an oven at 105 °C for 12 h. The percentage yield was then obtained from the latter.

### 3.2. Analysis of Total Phenols by Folin–Ciocalteu Reagent

The Folin–Ciocalteu reagent is used for the colorimetric determination of phenols and polyphenols. This reagent is a mixture of sodium phosphomolybdate Na_3_PMo_12_O_40_ and sodium phosphotungstate Na_3_PW_12_O_40_. The method is based on a redox reaction which leads to the formation of a blue chromophore, whose maximum absorption depends on the concentration of the phenolic compounds. It is detectable with a spectrophotometer in the range between 690 and 710 nm; in this research, a wavelength of 710 nm was used. A standard gallic acid (GA) was used to obtain the calibration curve, and the results for polyphenol content were expressed as mg/mL of gallic acid (GAE) [40].

### 3.3. DPPH Assay

The DPPH assay allows us to determine the antioxidant power by reacting the sample to be analyzed with a solution of DPPH [2,2-diphenyl-1-picrylhydrazyl] and analyzing the decrease in the radical peak under the visible wavelength at 517 nm. Antioxidant compounds (AOH), which are capable of transferring a hydrogen atom to the radical, cause a discoloration of the solution. The decrease in the peak at 517 nm of the radical (DPPH) after a pre-established incubation time (blank) is then analyzed under visible wavelength. This decrease (discoloration) is proportional to the antioxidant load present in the sample by applying the formula in [41].

### 3.4. FRAP Assay

Ferric reducing-antioxidant power (FRAP) assay represents another method by which to evaluate the antioxidant power. In particular, 0.1 mL of extract was added to 2 mL of reagent in acetate buffer (0.3 M, pH 3.6), 2,4,6-tris(2-pyridyl)-s-triazine (TPTZ) (10 mM) in 40 mM HCl and ferric chloride (20 mM) in a final ratio of 10:1:1 (*v*/*v*/*v*). After 30 min of incubation at room temperature, the absorbance was read at 593 nm. Similarly, a blank sample was prepared (prepared in the same way but without the extract). The unit of measure was the milligram equivalent of trolox per gram of dry extract (TEs/g of extract) [42].

### 3.5. Statistical Analysis

The results of each experiment were performed in triplicate and are presented as mean ± standard deviations (SD). Data were analyzed using multifactorial analysis of variance (ANOVA).

## 4. Conclusions

The study carried out on the selected plants had the aim of comparing the extract obtained from a mixture prepared with three plants in equal proportions (1:1:1) with that obtained by mixing the extracts of single plants in the same proportions to verify the efficiency of the two procedures.

To this end, two solid–liquid extraction techniques were used, namely, conventional maceration and the innovative RSLDE extraction technology.

The analyses carried out for the determination of the dry residue—the tests by Folin–Ciocalteu and DPPH—have enabled us to highlight that the extraction of the ternary mixture of plants is “richer” in bioactive substances compared to the mixture obtained by mixing the pure extracts in the same amounts. The probable explanation is that the higher values obtained via extraction in the mixture can be attributed to a “synergistic” effect due to the presence of other plants.

In the future, further studies will be carried out on the identification and quantification of the bioactive compounds present in the various types of extracts obtained.

Furthermore, from the comparison of the two extraction techniques used, it can be deduced that RSLDE, thanks to faster extraction kinetics and better efficiency in recovering the extract, could certainly replace the techniques of maceration, infusion and percolation.

Finally, it must be noted that the extraction of single plants, although less efficient, has its advantages; it allows us to obtain individual extracts to be used for the formulation of infinite blends, allowing the creation of different products in the food sector such as bitters and various drinks, as well as various types of supplements, and also in the pharmacological sector.
